# Spirit of the British and American Periodicals, &c.

**Published:** 1843-07-01

**Authors:** 


### Spirit of the British and American Periodicals.

---

### Strumous Peritonitis.

In the March Number of the Dublin Journal we have two Papers on this subject—one from that accomplished physician, Sir Henry Marsh—the other from Dr. Churchill.

Sir Henry Marsh's communication is chiefly occupied with cases of the disease. Dr. Churchill's contains a resumé of its history and management. To this we shall first and briefly allude.

The disease is an inflammation of the serous membrane of the abdomen, accompanied with effusion, and attacking persons of a strumous diathesis. It may occur either in an acute or chronic form, the latter being either a consequence of the former, or originating per se.

It generally occurs between childhood and puberty, or for a few years after that period—it is almost confined to children of a strumous habit and lymphatic temperament, and is often complicated with mesenteric disease.

*Causes.*—Bad diet, wet, cold, &c. may be enumerated. In most instances, it is hard to assign a cause. There may be extension of irritation from the intestinal mucous membrane—or it may follow febrile diseases.

*Symptoms.*—"The mode of invasion varies widely. In one class of cases the patient labours under diarrhœa for a considerable time with or without pain; the appetite is pretty good, the temperature natural, and the pulse quiet; but at length—it may be weeks or months—we hear complaints of a sensation of pricking, or of paroxysms of pain, and a feeling of tightness in the abdomen, which, upon examination, is found to be more or less swollen.

"In other cases there is a certain amount of pain from the beginning, occurring in paroxysms, with perfect intervals, and though at first limited to one part of the abdomen, yet by degrees spreading over and occupying the whole."

The early symptoms may be so slight as to escape attention, emaciation at last awakening it. There may or there may not be pain in the commencement. Sooner or later, however, this occurs most frequently in paroxysms of varying intensity and duration, with intervals of complete relief; beginning in some one part of the abdomen, and gradually spreading over the entire. There is generally tenderness on pressure, and the patient almost always complains of uneasiness on attempting to walk or stand, and in some cases finds it impossible to stand erect.

After an uncertain interval, fulness is complained of, and the abdomen will be found more or less swollen. Percussion generally yields a dull sound, but not always, for when the bowels are much disordered, they sometimes become tympanitic.

Fluctuation is generally distinguishable, if carefully made. According to Dr. Churchill, the best mode is to lay the child on its back, and accustom it for a short time to the presence of the hand upon the abdomen; then, placing one hand, with the fingers separated, on one side, and percussing very gently with the other, the muscles will not be excited into action; and, if fluctuation be perceptible with the second or third finger, we may be certain of the presence of fluid; for the pressure of the forefinger upon the skin effectually arrests the vibration which results from its elasticity.

The enlargement of the abdomen is not always equable—as it increases, the whole abdomen becomes tense and hard, with a hot dry skin, and large blue veins ramifying on it.

Sometimes the intestinal functions are long performed with regularity; but, in most instances, "we find the tongue white, loaded, and flabby; more or less thirst; the appetite irregular and fastidious, sometimes increased, more frequently impaired or lost altogether; the bowels relaxed or constipated, perhaps alternately; the stools fœtid and of a whity-brown or blueish colour."

As the disease advances the pulse generally ranges from 100 to 140, with pyrexia of the hectic character.

*Formations.*—It is a fortunate issue when *resolution* occurs.

The disease may end in a circumscribed collection of the effused fluid and its final evacuation, with more or less subsidence of the original affection. Under such circumstances patients have been known to recover. Dr. Burns mentions a case of this kind; and Dr. Abercrombie states that the matter may make its way through the abdominal parietes or the inguinal ring.

But *death* usually occurs, sometimes with unexpected rapidity, in consequence of ulceration and perforation of the intestines.

*Post-mortem Appearances.*—"Occasionally the vessels of the peritoneum are injected, though sparingly; there is more or less serum effused into the abdominal cavity, with shreds of lymph floating therein. The intestines are more or less agglutinated together, and often thus assume the appearance of sacs of matter. Where there has been perforation of the intestines, we find fæcal matter mixed up with the serum, and can generally detect the communication with the intestine through which it has passed. The peritoneum itself is often thickened, and coated with a layer of lymph; sometimes it is studded with miliary tubercles, or has tubercular matter deposited upon it. In some cases the mucous membrane is intact, in others, ulceration has advanced to different stages. The mesenteric glands may be free from disease, or they may be enlarged, and contain tubercular matter."

*Diagnosis.*—"When pain and swelling of the abdomen, with fluctuation, are present, the diagnosis will be easy; but in those cases in which there is no pain, and but slight tenderness, with little disorder of the digestive organs, there may be great difficulty. Our principal guide is the enlargement of the abdomen, which ultimately always occurs, and the fluctuation, which, by a little care, may generally be perceived. When there is much dyspnœa, or when the diarrhœa is severe, we must be on our guard against supposing the disease limited to the chest or mucous membrane of the intestines. We know that both may be seriously involved, concurrently with the peritoneal membrane. The same may be said of the mesenteric glands; they may also be diseased; but when they are affected alone, we shall find neither the abdominal swelling (at least to the same extent) nor the fluctuation."

*Prognosis.*—It is generally unfavourable. When the mesenteric glands, or intestinal mucous membrane, or pleura are involved, the case will probably end badly.

*Treatment.*—That usually recommended consists of leeches to the abdomen, fomentations, purgatives, of which calomel forms one of the ingredients, alteratives sometimes, tonics, chalybeates, absorbents, &c. But some cases are curable, if treated early and well. Dr. Churchill advises:—General bleeding, perhaps, never—leeches—poppy fomentations—a piece of lint wet with laudanum on the abdomen—a warm bath every, or every other night—castor oil, or Gregory's powder if necessary—astringents and anodynes for diarrhœa.

"But our principal reliance is upon mercury, given so as to affect the gums, if possible. I believe that the credit of thus administering mercury in this disease is due to Sir H. Marsh, as I have found no allusion to it in any authority. It may be exhibited internally or by inunction; in many cases the latter is preferable, as when diarrhœa occurs, the bowels are too irritable. A scruple of the strong ung. hyd. should be gently rubbed in over the abdomen, night and morning, and continued until the gums are touched, or the disease shows signs of yielding to the treatment.

"Blisters to the abdomen are very useful; they should be small, and applied successively to different parts, and dressed with the blue ointment."

On the subsidence of pyrexia, tonics and more generous diet—every precaution during convalescence.

Many cases are related by Sir Henry Marsh and Dr. Churchill. The preceding description rather refers to the chronic form of the disease. We subjoin a sample of the acute.

*Case.*—"A girl, aged 13, one of a family, every member of which exhibited signs of struma, was attacked with gastric remitting fever, presenting the usual symptoms of that disease. On the fourteenth day of her illness, an abatement of the febrile symptoms having taken place, she was suddenly seized with abdominal pains, rendered more severe by pressure. There was a great increase of fever, the pulse became exceedingly quick, small, and tense, the skin hot and dry, with an expression of extreme distress and anxiety in the countenance. The pains were relieved by fomentations, by the application of a few leeches (for at that stage of the fever, she could ill bear the detraction of blood), and by the internal administration of calomel and opium. Still, however, the general distress continued to increase, and, after the lapse of some hours, she presented all the characters of one dying of peritoneal inflammation: the abdomen began slowly and gradually to enlarge; it became tense, but not in the slightest degree tympanitic; the pulse was now so small that it could scarcely be felt, and so rapid, that it could not be numbered; the debility was extreme, and she appeared to be sinking fast. At the expiration of about two days, during which time her death was hourly expected, the abdominal distention had considerably increased, and fluctuation had become unequivocally perceptible. Mercury was rubbed in over various parts of the body, in quantities only limited by the fatigue of the patient, and was also continued internally. The bowels all the while yielded readily to injections, and the evacuations were fluid and bilious. After some time, all abdominal pain having ceased, and the abdomen having reached its highest degree of distention, the pulse became more distinct, and the debility less overwhelming; the skin began to relax, and slight mercurial ptyalism was established. The urine, which had hitherto been high-coloured and scanty, became more abundant, and deposited a copious lateritious sediment. The relaxation of the skin, the increased renal secretion, and the slight ptyalism, occurred simultaneously. Gradually the abdomen became less tense, and, as was proved by measurement, slowly diminished in size. During the progress of this slow process of absorption, the patient was nourished with farinaceous food and ass's milk. The influence of mercury on the system was still moderately maintained, and the febrile symptoms gradually receded. At the end of about twelve days the abdomen had subsided considerably. The patient daily improved in health, and ultimately no traces of the effusion remained. She is now, after the lapse of four years, restored to tolerably good health; the alvine functions are normal, but she looks pale and delicate, and requires, in her general management, more than ordinary care and attention. A few months since, having been attacked with catarrhal fever, which proved tedious and obstinate, the ingress of phthisis was seriously apprehended. She is now, however, exempt from signs of pulmonary disease, and enjoys as much health as falls to the lot of those whose constitutions are innately delicate and feeble."

We think that the profession are under obligations to Sir Henry Marsh and to Dr. Churchill, for directing their attention so decisively to this disease. And the stress laid on the use of mercury is likely to prove eminently serviceable.

---

### ON THE USE OF NITRIC ACID IN CERTAIN FORMS OF HÆMORRHOIDAL AFFECTIONS. BY DR. HOUSTON.

After all that has been written about piles, we believe that many medical men are anything but well-informed respecting them. They look upon them as varicose veins, and as nothing else—a capital mistake. Dr. Houston's paper, which we shall now notice, exposes this delusion.

1. The form in which hæmorrhoids most commonly exist, is that of a simple varicose state of the veins; and it would be well if to all such, the names "varices," or "varicose tumors of the rectum," were applied and restricted. We agree with Dr. Houston. Few persons, past middle life, are free from these. They appear and disappear on the mucous surface, or under the skin, in the neighbourhood of the anus.

*On External Hæmorrhoids,* of this character, we need not speak. They are sufficiently understood.

*Internal Hæmorrhoids.*—Dr. Houston believes that varyx is not the condition which most usually produces distress in "inward piles."

"The state of the mucous membrane covering such varices would appear to influence the condition of the case, more than that of the varix itself. While the mucous membrane continues smooth and pale, and free from morbid sensibility, there will be little distress; and the tumors will swell and subside, and even pour out blood occasionally, without the patient being at all aware of the extent of organic derangement which he labours under: but as soon as a relaxed state of the membrane,—a state to which it will soon be brought by the irritation and dragging to which it is subjected by the pressure of the fæces against the tumor of the varix, and the efforts of straining to overcome constipation,—as soon as this state of the membrane is induced, then, the varix coming down under the sphincter, is strangled, made to bleed, and to inflame. Or when, from similar causes, ulceration of the mucous membrane over the varix is established, then, distress of another, and even a worse description, viz., tenesmus, muco-purulent discharges, strainings at stool, and hæmorrhages of dangerous amount are entailed upon the sufferer. Or, still farther, when that state of the mucous membrane, to which the term "vascular tumor" is applied, supervenes, as I believe it often does, upon a varix, then, a simple and otherwise innocuous affection becomes one productive of most poignant suffering."

*Vascular Tumor.*—Dr. H. is disposed to regard this as an affection of the mucous membrane and sub-mucous tissue exclusively. Though it may be independent, it usually has for its basis a knuckle or bunch of varicose veins.

"I have seen it covering the surface of one varix in a rectum, while others in the same bowel have been smooth, and free from any such growth,—the former being the source of much annoyance, the latter giving no trouble at all. I have also seen the affection, in young individuals particularly, where the veins were quite free from any varicose dilatation, but in whom, after a time, varices formed as the result of the irritation of the vascular tumor. And I have observed that, in almost all cases of inward piles of long standing, no matter whether the affection have begun originally as a varix, or as a degeneration of the mucous membrane, both affections come to be present in conjunction, reciprocally aggravating each other's severity."

These tumors vary in size, and in number from one to so many as to cause, by their protrusion through the anus, a permanently widened state of that aperture, and a habitual prolapse, not only of the tumor itself, but also of a portion of the bowel. Dr. Houston's description of the tumor is graphic.

"In the early periods of the affection, the tumors are so soft, compressible, and free from pain as scarcely to be discoverable by the finger when introduced into the rectum, and scarcely, therefore, to be deserving of the term, tumor; but, when of long standing, and especially when they have been permitted to remain down for protracted periods at the water-closet, they acquire an increase of firmness and a tenderness which render them easily detected by such manipulations, and give them a tangible and permanent character. The dragging and pressure to which they are subjected in being pushed out and squeezed by the sphincter in defecation, renders them likewise prominent, and gives to them often a pedunculated or polypus-like form. The surface of the tumor is either granulated like a strawberry, or of a villous aspect. It is of a red colour, and, when protruded from the anus, bleeds from every pore as from a sponge. It is easy to satisfy one's-self on this latter head, by drying the surface of the tumor, when a fresh issue of blood instantly takes place from every point of its surface. The blood discharged, in such cases, is always of the arterial red colour,—a circumstance which often, in itself, indicates the true nature of the affection, and enables us to distinguish it from rupture of a varix. But, nevertheless, although this may be true as regards the direct issue of blood from the part, yet this very fluid, may, if allowed to lie in the cavity of the rectum before being discharged per anum, acquire a dark red, and even a grumous character. The bleeding in the former is also of more frequent occurrence, appearing with every effort at defecation, and weakening the patient more by the frequency and persistency of the drain than by the quantity lost at stated periodical intervals, such as usually takes place in the bursting of varices from over-distention."

Dr. Houston thinks that, under the head of "vascular tumors," there are two varieties of organic lesion, curable by the same means. One of these is that to which the term "erectile" has been applied, from the supposed resemblance of the disease to congenital affections of this class; the other,—a congested, hypertrophied, and tender state of the membrane, the result of irritative, or inflammatory action.

The first, he observes, "is regarded by many as a sort of aneurism by anastomosis of the small vessels of the mucous membrane and sub-mucous tissue exclusively, and may be independent from the first of varices of the general veins about the anus. This affection may occur in youth, and has been seen high up in the intestinal canal; but its most frequent seat is the lower part of the rectum. There is not originally or necessarily any pain arising out of it; but, by long exposure in the rectum to many sources of irritation, and by enlargement and prolapse from the anus, it runs into a state of actual disease, of which Cases I. and II. may serve as examples. It differs from ordinary nævi in not being necessarily congenital, but resembles them too much in its persistent tendency to increase in growth. They are, both, affections which equally require operations for their removal."

The second variety of vascular tumor is of a chronic inflammatory nature, and resembles the conjunctiva in old cases of chronic conjunctivitis. Like that, it secretes pus independently of ulceration.

"Such tumors are apt to form on old internal varices, which, by their projecting into the cavity of the bowel, expose the membrane covering them to more than ordinary pressure and irritation, and thereby become the direct cause of this morbid development. Once established in connexion with the surface of a varix, the two making between them a compound disease, the distressing qualities of such an affection are not slow in exhibiting themselves. Thus, in case No. III., in which there were several internal projecting varices, that only on which this hypertrophied condition of the mucous membrane existed, gave annoyance, and on the removal of that and that singly, all hæmorrhoidal distress subsided. The 'master pile' being removed, the others fell back into a state of painful quiescence. As in the foregoing variety, there is no relief for this affection but in destruction of the morbid growth."

If this be true, asks Dr. Houston, why use knife or ligature for a disease of surface? He advises for it the *pure nitric acid,* and apply it in the following way.

"Let the patient strain as at the night-chair, so as to bring the tumors fully into view; and while they are so down, let him either lean over the back of a chair, or lie down in the bent posture on the side on which the disease exists, with the buttocks over the edge of the bed. Let a piece of wood, cut into the shape of a dressing-case spatula, be dipped in the acid, and then, with as much of the acid adhering to it as it will carry without dripping, let it be rubbed on the tumor to the extent desired. The due effect of the acid on the part is shown by its changing it to a greyish-white colour. If a superficial slough be all that is required, a single application may be enough: if a more deep one, then, two or three applications of the wood dipped in the acid may be made in quick succession; which being finished, let the part be well smeared over with olive oil, provided beforehand for the purpose. The prolapsed parts should then be pushed back within the sphincter, the patient put to bed, and an opiate administered. The pain of the application is sharp, and burning at first, but goes off in two or three hours, and does not again return in the same form. A general uneasiness about the anus, on motion, together with a slight sense of heat, fullness, and throbbing, are felt for a few days; and there may be some little feverishness; but I have not seen or heard of any more serious effects from the remedy. In Case II. a slight stranguary, which was experienced for a short time, disappeared under the mist. camphoræ c. opio. The symptoms following the application of the acid are usually so mild as not absolutely to require confinement to bed more than a few hours; although, for many reasons, such confinement may often be desirable. On the third or fourth day, a purgative draught should be administered, when the bowels will be found to yield to the medicine, generally without either pain or prolapse of the rectum. The progress after this to healing is rapid, and free from any disagreeable symptoms."

We shall certainly make trial of Dr. Houston's pıoposal.

---

### ON THE USE OF OIL OF TURPENTINE IN HEMERALOPIA OR NIGHT-BLINDNESS. By CHARLES KIDD, Esq.*

Two cases of this rare disease came under Mr Kidd's care in the latter part of 1842. Nothing abnormal could be detected about the eyes in either case, with the exception of some sluggishness in the contraction and dilatation of the pupils, with a general heavy appearance about the forehead and eyes. After employing the ordinary treatment by aperients and tonics without any benefit, the iodide of potassium, mercury, succession of blisters, strychnine, &c. were tried but without making any impression on the disease. At last, considering the imperfect action of the pupils, the want of the usual dilatation at night, perhaps shutting out a large proportion of the faint rays which would otherwise get through the lens to the retina, Mr. Kidd was led to employ turpentine, a medi-

* Dublin Medical Press, May 10, cine possessing great influence over the iris. In the course of a fortnight after the use of this remedy, the first case was discharged cured, and shortly afterwards the second became entirely relieved of this distressing complaint, and has continued well up to the present time.

Mr. Kidd remarks, in conclusion, "from the rapid effect of the ol. terebinthinæ in these cases, I am quite satisfied of its specific action being exerted on the *muscular* tissue, shall I say (?) of the iris, or perhaps on some *serous* accumulation under the membrane discovered by Dr. Jacob. The latter more than once struck me as its *modus operandi*, but of course it would be premature to give an opinion from two cases. If it should on further examination prove to be the case, it is only an additional instance of how deeply indebted we are to the labours of that eminent anatomist."

---

### On the Special Function of the Skin. By R. Willis, M.D.*

Though the due performance of the function of the skin is well known to be of great importance to the health, physiologists are not yet agreed as to the precise purpose in the animal economy which it performs. Dr. Willis considers the water as the essential excretion; not, however, in the usual sense in which the word excretion is used, nor yet as a means of regulating or reducing temperature. In what way then does it happen that health and even life can be immediately dependent on the elimination from the general surface of the body of some thirty-three ounces in the course of the twenty-four hours? To this the author answers,—by securing the conditions which are necessary for the endosmotic transference between arteries and veins of the fluids that minister to nutrition and vital endowment.

It is admitted by physiologists that the blood exerts no influence on the body until that portion of it which has been denominated the plasma has transuded from the vessels and come into immediate contact with the particle that is to be nourished and vivified; but no physiologist has yet pointed out the efficient cause of these tendencies of the plasma, first to transude through the wall of its efferent vessels, and secondly to find its way back again into the afferent conduits. The explanation given by the author is, that in consequence of the out-going current of blood circulating over the entire superficies of the body perpetually losing a quantity of water by the action of the sudoriparous glands, the blood in the returning channels has thereby become more dense and inspissated, and is brought into the condition for absorbing, by endosmosis, the fluid perpetually exuding from the arteries, which are constantly kept on the stretch by the injecting force of the heart.

Repeated experiments have demostrated the fact that the blood in the veins is of somewhat greater density than that in the arteries. If the specific gravity of arterial blood be allowed to be 1050, that of venous blood in the mean will be 1503.

In an appendix to the paper, the author points out a few of the practical applications of which the above-mentioned theory is susceptible.

In temperate regions, interference with the function of the skin, and principally through the agency of cold, is the cause of the greater number of acute diseases which prevail. Animals exposed to the continued action of a hot dry atmosphere die from exhaustion: but when subjected to the effects of a moist atmosphere of a temperature not much higher than their own, they perish by the same cause as those which die from covering the body with an impervious

---

* Medical Gazette, April 14.

glaze; for, in both cases, the conditions required for the access of oxidised, and the removal of de-oxidised plasma are wanting, and life necessarily ceases. The terms *miasma* and *malaria* may, according to the author, be regarded as almost synonymous with air at the temperature of from 75° to 80° Fahr., and nearly saturated with moisture.

The secretion of the skin otherwise suppressed is attended with no less fatal consequences. What kills a patient within the first 48 hours of a bad attack of scarlatina? The autopsy shows nothing amiss. He died undoubtedly from the complete suppression of the function of the skin.

---

### AMMONIACAL URINE.

In one of the excellent lectures on the physical and pathological characters of Urinary Deposits, by Dr. Golding Bird, now being published in the Medical Gazette, in speaking of Carbonate of Lime, Dr. Bird takes the opportunity of expressing his doubt whether the urine is by any means generally *secreted alkaline*, even in those cases of alkaline urine produced by blows on the back, &c. Urea may be regarded as carbonate of ammonia, *minus* the elements of water, or a *carbonamide*. The conversion into the carbonate takes place but slowly in healthy urine out of the body, and still less frequently whilst retained in the bladder. Under certain circumstances, however, especially where an excess of mucous matter is secreted, this substance acts the part of a ferment, induces a new arrangement of the elements of urea and water, and thus the urine is voided in an ammoniacal condition. Under ordinary circumstances the vital powers of the bladder are sufficient to prevent the urine from undergoing this change, even though much mucus be present. When, however, the nervous influence is materially diminished, as in cases of injury of the spine, &c. the contents of the bladder become subject to the ordinary laws of chemistry. In this instance, Dr. Bird is induced to believe, from the result of many cases, that the urine is secreted by the kidneys in the usual subacid condition, and that it becomes alkaline by the subsequent change which it undergoes in the bladder, when the vital endowments of the latter have become depressed, and the mucus acting as a ferment converts the urea into carbonate of ammonia.

Thus in the case of a woman labouring under complete paraplegia, whose urine was seldom voided, and was ropy, alkaline, and offensive, on washing out the bladder with warm water, and leaving an elastic gum catheter in the urethra, so that the urine might escape almost as soon as it reached the bladder, the urine appeared clear, pale, and slightly acid, whilst that voided 24 hours before was dark, offensive, alkaline, and turbid, not only from mucus, but from sediment consisting of carbonate of lime and the earthy phosphates.

The best and most satisfactory cases in which the urine was secreted alkaline, are those recorded by Dr. Graves of Dublin; one of them was a case of adynamic typhus fever, in which an abundance of carbonate of ammonia existed in the urine, and a corresponding deficiency of urea and uric acid prevailed. As the patient became convalescent the carbonate of ammonia vanished, being replaced by an equivalent proportion of urea and uric acid. The second case was one of fatal general anasarca, following exposure to cold; here the urine was loaded with carbonate of ammonia, but neither urea nor albumen existed. After death the bladder appeared perfectly healthy.

---

### Some Cases of Painful Affections of the Fifth Pair of Nerves. By John Hamilton, M.R.I.A.*

Mr. Hamilton relates seven cases, all of them possessed of interest. We shall give a brief account of some of them.

Case 1.—*Painful Subcutaneous Tubercle in the Temple, over the Temporo-auricular Branch of the Superior Maxillary Nerve.*

Charles Dale, æt. 25, a shoemaker, "complains of the most distressing attacks of pain shooting from the temple all round the right side of the head up to the vertex, behind to the occiput, and before to the centre of the forehead; from the same spot proceed darts of pain like fire, to the right eye, with an appearance of red spots before the eye, and dimness of vision, and at the same time he gets deaf of the right ear, the eye becomes swollen, and there is such discoloration about it that he has often been asked if he had a black eye; the pain also engages the side of the face, but never the jaw or teeth. There is a general soreness of the whole scalp at that side; but he refers the origin of all his sufferings to the presence of a small tumor at that part of the temple from which the painful sensations spring; and on examination I discovered in front of, and just above the helix of the ear, at the roots of the zygoma, where the temporal artery commences, a small tubercle about the size of a grain of shot; it was moveable, and evidently rather deeply situated, and after a little handling disappeared, as if it sunk into the substance of the parts beneath: after he had performed the action of mastication, it became again apparent. He was always sensible of a painful feeling in it, increased by pressure or by the action of the temporal muscles in eating. The shocks of pain commenced like the blow of a heavy stick at this particular spot, and at such times the tubercle used to become larger. He has suffered from the complaint for eleven or twelve years; it began with paroxysms of pain about once a month, sometimes oftener, sometimes with intervals of three months, increasing in violence, at length he got an epileptic fit, commencing with a gushing sensation (aura epileptica) from the site of the tubercle (of whose existence he was not then aware), with insensibility and convulsions; he had eighteen of these fits, the last well marked one about seven years ago. Turpentine, which he then took, subdued them, and has since kept them in check, but not entirely, as he had shocks afterwards as nearly like the fits as possible. But during the whole of this long period, he has never ceased to suffer from occasional attacks of pain, which latterly have increased both in severity and frequency; so much so, that a day now scarcely passes without a shock, and in the interval he is never free from a dull, heavy, stupifying pain in the right side of the head; he has also, very lately, had some attacks bordering on epilepsy, short intervals of insensibility, preceded by an aura from the tubercle." His general health was bad—his symptoms were of a highly nervous character—and medicines had proved useless. Mr. Hamilton performed excision of the tubercle.

"An incision was made over the tumor, and a small dark-coloured tubercle became apparent, which was readily removed; but after its removal pressure, on the sides of the place where it had been, gave rise to disagreeable sensations, similar to what he had felt when the tumor had been pressed. I therefore removed two little substances in these situations, which, afterwards examined with a magnifier, had the appearance of portions of nerve. The tubercle itself was

* Dublin Journal, May, 1843.

round, of a brown colour, composed of a perfect cyst, containing a small, hard, calcareous substance of a chocolate colour; the surface of the tubercle was quite clean, nothing like nervous substance attached to it."

It was found to be composed chiefly of carbonate of iron, with a little carbonate of lime.

For some days after the operation, he was free from his former sensations, and his health improved remarkably; but in the course of a fortnight the old symptoms returned. "On examination, I found a small moveable tumor in the situation of the other; I was puzzled whether this was a second tubercle or not. Cutting down on it, I could discover nothing definite; there only appeared a little whitish condensed cellular tissue, which was painful on pressure: this I took as my guide, removing every part of the substance that produced the peculiar sensation on pressure. When I had done so, to some depth, I clearly distinguished a branch of a nerve crossing the bottom of the wound; touching this with the blunt end of a probe, induced a peculiar uneasy feel, which made him exclaim that he was sure I was touching a nerve. I now understood the second accession of painful symptoms to have been caused by the pressure of the cicatrix (formed after the first operation) on this nerve; the cicatrix occupying the situation of the tubercle, and producing the same mechanical effects. As there appeared to be some morbid sensibility of the sides of the incision, I touched them lightly with caustic kali, and dressed the wound from the bottom."

It is now eight months since this operation, and the patient continues well.

Mr. Hamilton observes that the nerve affected was probably the temporo-auricular, or temporalis superficialis of the superior maxillary branch of the fifth. The tubercle itself differed in structure from the common subcutaneous tubercle, which is usually fibrous, fibro-cartilaginous, or cartilaginous. The most analogous case is reported by Mr. Windsor, in the Edinburgh Medical and Surgical Journal for 1811; he removed a tubercle from the forearm of a woman, which proved to be a cyst, containing numerous little grains, which gave the sensation of sandy or earthy bodies.

Mr. Hamilton knows but of one other case in which subcutaneous tubercle was met with in the head. That case occurred to Sir P. Crampton. But he has seen an instance of it on the inside of the right side of the upper lip.

CASE 2.—Catherine Dunfield, æt. 25, had had strumous sore throat, and superficial lupus on the forehead, as well as painful sciatica.

"May 1, 1840.—She went to bed well, but awoke in the night with a most severe pain in the back of the head, and darting through the ears. It was most acute, and lasted till morning. I saw her a week after. During the intervening time she had had each night a paroxysm of the pain, which went off towards morning. She had a second paroxysm at three o'clock, P.M. and suffered from it at intervals during the day. The attacks were increasing in severity. She was nervous and low. Pulse 76; tongue slightly furred; no sickness of stomach; bowels, confined.

"Purging, quinine, mercury to slight salivation, colchicum, a small cupping at the back of the neck, and a blister to the occiput failed to relieve her; but on the contrary, she became much worse, particularly after the cupping, though only four ounces of blood had been removed. She had hitherto been up, but on the 11th I found her in bed, and she said that the night before she thought she would have gone distracted with the pain. The pain had also changed its situation, having extended to the vertex, right temple, cheek-bones and side of face, and the upper part of the side of the neck; in short, it engaged the temporo-facial, facial, and cervico-facial branches of the portio dura of the seventh pair of nerves.

"All these parts were tender to the touch. I had asked her before whether there was any sore on any part of the head, and had been contented with her assurance that there was not. I now, however, made a careful examination, and found at the right side, about the upper and posterior part of the parietal bone, a small scabby crust; when this was removed, there appeared a small lupoid ulcer, the size of a fourpenny, deep, undermined beneath the edges, the circumference tumid and tender. The true nature of the case now struck me; the present ulcer had gone deep enough to affect the pericranium, and at first one of the posterior superior branches of the seventh nerve, and the branches of the occipital, by the free communication which exists between them; that in a less irritable temperament, the effects of the ulcer might have been purely local, and confined to that part of the nerve engaged in the ulcer; but in her nervous excitable constitution, the morbid irritation had extended to all the ramifications of the nerve. As it was obvious that the only treatment likely to afford relief, was that directed to the ulcer, I made a transverse incision through it down to the bone. That night she first got ease, freedom from pain, and sleep. She had no return of the pain, and soon got quite well."

Case 3.—Ellen Whelan, æt. 40, mother of eight children, suffers dreadfully from pain in the left side of the face, beginning at the upper jaw, above the molars, and going up the side of the head and out through the ear. It generally gets worse at 11 at night, and keeps her awake till day break. Is rather deaf in the left ear—has had the pain in the face for the last four months. Has taken much medicine, been blistered, and had several teeth drawn without benefit.

"Examining the left ear, I found it blocked up with wax, of extreme hardness. This I removed, not without some pain, and afterwards syringed the ear well. Immediately after this she fainted, and remained in a state more nearly resembling death than I had ever seen before, for half an hour.

"After recovering from this, not only the deafness, but *all pain in the side of the head and ear, had disappeared.*"

That night she slept well. Next night the pain in the jaw returned. Under blue pill and purgatives for a few days, followed by half a drachm of bark and a scruple of carbonate of iron three times daily, she got quite well in a week.

Mr. Hamilton gives a case of what he considers acute inflammation of the fifth nerve, set to rights by leeches, blistering, and calomel and opium. This leads him to chronic affections of the fifth, or tic douloureux. On this he remarks:—"It has lately been recommended, much too exclusively I think, to combat the disease by what has been called rational constitutional treatment, directed to improve the gastric and hepatic derangements; which derangements it has been attempted to prove are the causes of the majority of what are called idiopathic cases of tic doloreux. Were they really so, this treatment would fail much less frequently than it does. But those who see many examples of painful affections of the fifth or other nerves will allow that in the greater number of instances they cannot be traced to gastric derangement, and that in those cases where the stomach and liver offer much functional disturbance the removal of this does not always, or even generally, cure the neuralgia. In many cases, on the other hand, the gastric derangement, and the general disturbance of most of the organs in the system, follow the pain in the nerve, and in such cases the tongue frequently cleans under the use of opium. Often, however, treatment directed to improve the secretion of the liver and bowels, is the best preparation for tonics or narcotics, and lays the best foundation for a complete cure. Where I conceive this plan most suitable I generally give three or four grains of rhubarb and as many of blue pill at bed-time, with the sulphate of magnesia, sulphuric acid, and infusion of roses, the next morning, for three or four nights and mornings; after this the patient feels better generally, but the pain is usually but little affected. I now give a drachm of bark and a scruple of carbonate of iron, three times a day; and where the pain becomes more severe at night, two grains of the extract of hyosciamus and one of opium, at bed-time, to be repeated in the middle of the night if necessary. This treatment very often succeeds, much more so than beginning with bark, quinine, or carbonate of iron, at once, without any regard to the state of the digestive organs. Local depletion, particularly in the florid, and blistering, are very useful; but in most chronic cases the abstraction of blood aggravates the pain. There are cases, however, in which it is only wasting time, and prolonging the patient's sufferings, not to begin at once with tonics and narcotics, which often effect a rapid and almost unexpected cure."

He details three cases, but the observations are sufficient.

---

### OBSERVATIONS ON SOME MORBID AFFECTIONS OF THE NAIL OF THE GREAT TOE. By A. COLLES, M.D.*

Any observations on practical subjects from Mr. Colles must command attention. The following merit it.

1. "*Nail growing into the Flesh.*"

Mr. Colles alludes to the barbarous operations recommended and practised for this painful affection. His object is to shew that they are unnecessary. He first describes the disorder. We observe, he says, at the angle of junction between the anterior and external edges of the nail, and ulcerated fungus, into which this angle, as also a portion of the outer edge of the nail, are more or less sunken. The colour of the fungus is rather florid, its surface is smooth, the discharge is purulent, in small quantity and tolerably healthy, unless the part have been irritated by too much exercise of the limb or by some external application or local injury; there is little or no surrounding inflammation, no enlargement of the toe, and the pain is in general trifling, unless during exercise, when the weight of the body on the limb causes the nail to press into the soft substance of the fungus, which thus often induces considerable uneasiness and lameness.

The disease evinces little or no tendency to involve the adjoining parts. Dr. Colles doubts, as do we, its disposition to run into malignant onychia.

Passing over the enumeration of various methods of treatment, we give Dr. Colles's own:—

"I proceed as follows: while an assistant with a spatula presses down the fungus, I seize with a forceps (with strong flat blades, like those of the torsion forceps) the edge of that portion of the nail which is to be removed. I then pass a probe with a thin flat extremity beneath the nail, close to the fungus, as high as it can go, taking care to direct it towards the outer edge of the nail; this enables me to judge how far the nail is detached. I then take a strong crooked scissors of a large size, with one sharp pointed blade, this I introduce beneath the nail as far as the probe has directed me; with one stroke of the scissors I then cut off all this detached portion of the nail, while by means of the forceps I draw it away with moderate force; should this attempt fail to remove it, I then re-examine the part with a probe, and again introduce the scissors as high as they can be pushed; a second cut will then complete the separation, and admit of the easy removal of this part of the nail; this second attempt is sometimes attended with a sharp momentary pain, as the point of the scissors often enters a short

---

* Dublin Journal, May 1843.

distance into the sensitive matrix. The portion of detached nail presents no change of texture whatsoever; but a few drops of blood follow this operation. The only dressing required is a small bit of dry lint which is to be pressed firmly with the probe between the fungus and the edge of the nail. In a few hours the toe is free from pain, and the patient can walk without any lameness or uneasiness in three or four days after the operation. The dressing continues perfectly dry, and needs not be changed until the fourth day; at this time the fungus will be found much reduced in size, perfectly dry, and of a firmer consistence: a small bit of lint is to be re-applied as before, it should not, however, be pressed down, so firmly as at the first dressing. In the course of ten or fifteen days the fungus will have entirely disappeared, and the parts be restored to a healthy state. I have never found it necessary to apply the olive-shaped cautery to destroy the fungus, as described by Dupuytren. In the course of this treatment I also advise the patient (in compliance with the rules laid down by authors) to scrape the upper and outer surface of the nail with a sharp penknife, or with a bit of glass, an instruction, however, which the patient neglects, as soon as he feels himself relieved from pain.

"It affords me much gratification to be enabled to state, that in no instance have I met with a case of relapse, after this operation has been efficiently performed. But the result of the operation itself is not in all cases so successful as I have hitherto represented, for in some instances, four or five days after the operation, the patient will complain of some uneasiness in the toe, when we shall find, on examination, that the dressing is moistened with a little discharge, and that a small portion of a whitish substance, like soft and swollen leather, is rising up through the fungus. This substance, may be, is regarded as a sort of accessory ungual filament, arising close to the original nail from the anterior and outer border of its matrix, and which is now altered in texture and direction; this filament is so soft, that it breaks and tears, if caught by the common dissecting forceps; in order, therefore to remove it (which it is necessary to do), we must seize it with the torsion forceps, and excise it with one cut of the scissors, passed well and fully beneath it; the lint dressing is to be reapplied, in the manner before mentioned, and the case will proceed without further interruption to a perfect cure."

We have been for some years in the habit of adopting a nearly similar proceeding with complete success. But there are some slight differences between our method and that of Dr. Colles.

Supposing the fungus to surmount, as it generally does, the border of the nail, we first touch it freely with the nitrate of silver. Then, having previously had the nail well soaked and pared, we insinuate under it the flat end of an eyed probe, and turn it up. If that can be effected we cut off the reverted edge with a proper pair of scissors, and proceed with the probe and forceps to remove all the remainder of the nail which covers the unsound matrix, or fungus, and is *detached*. If the edge of the nail is not turned up by the probe we gently introduce a piece of lint beneath it, and leave it until the following day. The nail being again soaked and scraped, the portion over the lint can now be cut away, and a fresh portion of lint introduced farther under the nail, while, if necessary, the fungus may again be touched with the caustic. By proceeding in this way the portion of the nail which interferes with the fungus may in two or three days be removed, with scarcely the slightest pain to the patient. The caustic is advantageous in two ways—first it destroys or greatly diminishes the sensibility of the fungus—and, secondly, it destroys its projection which would interfere with the other parts of the treatment.

If the nail is very loose we may proceed in Dr. Colles's manner. This we have also done on several occasions.

Dr. Colles goes on to state:—

"There is another morbid affection, which occasionally engages the anterior and inner angle of the great toe nail, and which causes considerable lameness and uneasiness, particularly on pressure; this affection is often mistaken for an attack of gout, particularly in those persons where such an attack may be expected, or even desired. In this disease there is no swelling or redness; but pain, on pressure, at the anterior and internal angle of the nail. On a close examination of this spot, we find that this angle rests on a hard, white mass of laminated, horny cuticle, which we can easily remove in bran-like scales, when we shall see a small cup-like cavity, without any ulceration or disease. The ungual angle appears thick and bulbous opposite this point, and the pain is caused by its pressing against this mass. This affection is easily cured, by scraping away all this substance, and excising the bulbous angle of the nail, and then interposing a little lint. Attention is required, for some little time, to remove any unhealthy growth of the cuticle or nail, and to secure the patient from any further uneasiness. Finally, I may remark, I have never seen this disease engage the outer angle, neither have I seen that last described engage the inner angle of the toe nail."

2. *Onychia Maligna.*

Dr. Colles gives a succinct, but good account of this severe disease. This, however, we omit. He goes on to say, that all modern authors concur in representing as indispensable the removal of the diseased matrix. He observes:—

"I admit that the operation of the complete removal of the entire of this diseased matrix does effect the cure, in a very short space of time, provided the bone or joint is not diseased (in which case, amputation is inevitable), and that, subsequently, rest and simple dressing will alone accomplish the healing process, the place of the nail being supplied by a dense, hard skin. But still this operation is not without objection; it is always attended with severe pain, and which sometimes continues for many hours; it also too frequently happens, that the disease returns in some one spot or other, owing to the matrix not having been wholly eradicated, which indeed it is often extremely difficult to do; for the shape of the toe is so bulbous, and so deformed, the textures so changed and so condensed by chronic inflammation, and the edges of the ulcer are so raised over the part to be removed, that even an anatomist cannot easily recognise the relations of the several tissues involved in the disease, or ascertain the exact extent of the substance to be excised: during the operation also, the flow of blood is so profuse, as to prevent the operator distinguishing one texture from another; accordingly, it occasionally happens, that the patient, having enjoyed an immunity from pain for some days after the operation, becomes alarmed by feeling a slight return of his former uneasiness, on any exercise of the limb, or on any pressure on some particular spot, generally on one of the angles of the original ulcer; and the surgeon, on a careful examination of this spot, finds there is still a little ulceration, and a fresh production of that ungual growth, already described as abnormal both in texture and in direction, and which indicates the persistence of some of the diseased matrix. This will again act as a foreign body, exciting constant irritation, and will soon lead to a renewal of all his former sufferings, and for the removal of which, the excision of this morbid tissue must be repeated. Any surgeon who has experienced this disappointment can, I am confident, bear testimony to the horror with which the patient has contemplated, and the reluctance with which he has submitted to, a repetition of this peculiarly painful operation. I have known it necessary to repeat this even a third time; and therefore, whenever these secondary operations become necessary, I should remind the surgeon, that, to insure success, he must cut much more deeply and extensively than he might at first suppose to be necessary."

Dr. Colles, however, thinks that the disease is curable in a less severe manner. The following plan has been in his hands, as yet, invariably successful.

"I confine the patient to bed, and direct a poultice to the toe for two or three days; I then cleanse the ulcer carefully, by directing on it, from some height, a small stream of tepid water from a sponge; I next cut away as much of the loose nail as I can, without paining or irritating the sensitive surface around, and then I fumigate the part by means of the mercurial candle, containing ʒj. of the hydr. sulphuretum rubrum (olim cinnabar), to two ounces of wax. This fumigation is to be applied night and morning, and, after each, the toe should be gently enveloped in lint or linen, lightly spread with ung. spermaceti. In four or five days the patient will express himself as considerably relieved; the discharge from the ulcer will be found of a healthy, purulent character, and the appearance of the whole part much more favourable. The fumigation is still to be persevered in, and all projecting portions of nail to be closely cut: I consider this latter direction as very essential, as thereby the mercurial fumes can have more free access to the surface of the ulcer. In proportion as the ulcer improves it is interesting to observe so does the condition of the growing nail; it acquires not only its natural firm and horny consistence, but also assumes its proper horizontal direction. For some time after the general surface of the ulcer has been healed, there still remain small spots of ulceration, generally at the angles, around some white germs of new nail; against these points, the full force of the mercurial vapour should be directed; this can be effected by adding a small conical ivory tube to the funnel. I attribute much of the success of this treatment to the use of the mercurial candle, in preference to fumigation in the ordinary mode. I must observe, that during this course of treatment, the patient must absolutely abstain from walking, or even standing on the affected limb; exercise but for a single day will counterbalance all the amendment produced by a week's rest and fumigation. In the cases which I have thus treated, I have had no recourse to any constitutional treatment, or to any peculiar regimen; no doubt, cases must occur in which there will be derangement of the general health, and which will require suitable constitutional remedies, before we can expect to cure the local disease by mere local applications. I make no doubt that this ulceration may sometimes arise from, or be so intimately connected with some constitutional derangement, as to be incurable, without the aid of constitutional treatment. Mr. Wardrop, in his paper above alluded to, has recommended a cautious and judicious use of mercury, and has recorded some instances of its success."

In a case of onychia maligna lately under our care in the hospital, we effected a complete cure, although the last phalanx was bare, by means of sarsaparilla and the oxymuriate of mercury, conjoined with poultices of bread and the aqueous solution of opium. We believe that in many cases constitutional and soothing local treatment will be successful.

---

### The Zoist.

Such is the title of a new Journal, "a Journal of Cerebral Physiology and Mesmerism, and their applications to Human Welfare." How the "Cerebral Physiologists" will like the union, we will not undertake to say, but we suspect that the better and the larger portion of them have little stomach for the match. The "Zoist" is ornamented with a neat vignette representing a venerable man, intended, no doubt for Dr. Elliotson, poring over a volume opened on his knee, while two females of prepossessing mien, but remarkably loose habits, support the Doctor upon either side, and compose a striking and interesting group. The ladies, of course, are the Okeys, and though we are aware that such gifted individuals are far above what are vulgarly considered the decencies of life, yet we would venture, with great diffidence, to hint, that their petticoats are *rather* scant.

By a singular coincidence, this Journal of Mesmerism starts upon the first of April.

The Prospectus informs us that the Editors of the Zoist "will endeavour to furnish a medium for the freest expression of thought on questions of social, moral, and intellectual progress. In giving an opinion on important questions, they will not be influenced by external movements, either popular or the reverse. They claim perfect independence of thought, but will be guided in the expression of it by the unerring principles of their science. They aim to be truth-seekers, and they consider it to be their duty to promulgate the 'truth of facts,' impelled by the conviction that all truths are subservient to the happiness of mankind." They talk it bravely, but "fine words," says the proverb, "butter no parsnips." The "unerring principles" of Mesmerism are, perhaps, appropriately employed to guage the independence of thought, themselves being independent of everything like thought. But to proceed:

"The discovery of a new truth gives to the philosopher intense delight. The science of MESMERISM is a new physiological truth of *incalculable* value and importance; and, though sneered at by the pseudo-philosophers of the day, there is not the less certainty that it presents the only avenue through which is discernible a ray of hope that the more intricate phenomena of the nervous system,—of Life,—will ever be revealed to man. Already has it established its claim to be considered a most potent remedy in the cure of disease; already enabled the knife of the operator to traverse and divide the living fibre unfelt by the patient. If such are the results of its infancy, what may not its maturity bring forth? Let us pause for a moment to survey our position. An acute susceptibility of pain has been diffused throughout the human body to warn man of injury, and hitherto it has been deemed a necessary consequence that suffering should follow the violation of its integrity. Behold, in a painless operation during the trance, the arrest of an important function at the will of man! and for the purposes of beneficence,—a result which a few years since it would have been considered madness to conjecture. Is not this a triumph justly deserving the name of glorious, quickening the pulse in the bosom of philanthropy, and unfolding bright visions of the future to the gladdened eye of the well wisher of his race? Shall *an era occur in the progress of man*, and the tidings fall on the cold ear of apathy and indifference?"

It is quite clear that the Zoists intend to be fine writers. It is not often, in these dull times, one meets with anything so inspiriting as the wind up of this passage. Visions and eras and gladdened eyes and cold ears all in five lines, are not things to be sneezed at. The mode of address, however, is more taking than novel. The "Behold" &c. is evidently an imitation of the apostrophic or show-man style. "Valk up, ladies and gentlemen, valk up, and see this ere vonderful halligatur, vot measures fifteen feet from the snout to the tail, and seventeen feet from the tail to the snout." The showman certainly may complain of plagiarism, but after all, *his* wonders are not fit to hold a candle to the Zoist's, for the world scarcely dreams of what is coming.

"The extraordinary phenomena elicited during the excitation of the cerebral organs by means of Mesmerism, and establishing a connection between the two sciences, constitute another department demanding the most serious attention and investigation, and the ultimate results of which may transcend in value and utility all that man has yet dared to hope for from science. Let what many facts render probable be once established, viz., that this state of increased activity can be rendered permanent and carried into the natural state, and who does not catch a glance of a mighty engine for man's regeneration, vast in its power, and unlimited in its application, rivalling in morals the effects of steam in mechanics."

Steam! Baugh. A trip to China and back in an afternoon by the aerial carriage is nothing to this. Look at its applications. We take a country bumpkin—make some passes attractive, repulsive, and centrifugal, and winding up with a few corkscrew touches over his bump of constructiveness, we qualify him architecturally to that degree, that Brindley would dilute into "treble skimm'd sky blue" beside him, and he might produce an impromptu of a pump that would astonish even Mr. Pecksniff. So, with a little management, we could change a poltroon into a hero, a booby into a poet of the first water, and in short "draw out" their talents from persons who never had any. Extraordinary science, fit to be ranked with astrology and alchemy, and the gentle craft of palmistry, all doubted as they are and snubbed in these unphilosophical days.

The "Zoist," if he does not aspire to the crown of martyrdom, anticipates it: "The science of Cerebral Physiology experienced strong opposition before even its first principles were recognized, or the truth of its most obvious facts conceded. The science of Mesmerism has been destined to pass through the same ordeal. There is one curious and melancholy feature which deserves especial notice, and which proves how little Cerebral Physiologists have profited by the difficulties they encountered during the infancy of their own science. Men of science denounced Gall as an enthusiast and visionary, and proclaimed his facts to be fallacious. Men of science and even Cerebral Physiologists denounce him who is convinced by his senses of the truth of Mesmerism as an enthusiast and visionary, and proclaim the extraordinary phenomena which are developed in the trance to be gross impositions. Thus the very course which Cerebral Physiologists condemn in the opponents of their own views, many of them are the first to adopt on the proclamation of a new and startling truth; and this is the more extraordinary, because many of the phenomena illustrate in a most beautiful manner the excited action of the cerebral organs. How great the difference between theory and practice! They believe in a philosophy which teaches them an opposite course of conduct and yet feel not its influence!

"However, those who are investigating nature and recording facts know that in a short time opposition must cease, and they turn from the gloomy to the bright side of the picture and contemplate in the distance the results of their labours. They look for their reward in the plentiful harvests of the future, rather than in the reapings and gleanings of the present or the past. 'Glorious, heroic, fruitful for his own Time and for all Time and all Eternity, is the constant speaker and doer of truth.' The assertor of truth may be crushed and we may breathe a sigh over the martyr as he passes from the field of his labours,—ignorance and prejudice may for a time reign triumphant, and the abettors of sloth and selfishness be considered the great, the good, and the wise,—but Time rolls on, and Reason will assert her dominion."

It certainly does seem, at first sight, ungrateful on the part of the phrenologists, to disbelieve in Mesmerism, seeing what disbelief was long extended to themselves. But this may be said in their defence, that if the mere circumstance of having met with incredulity, is to constitute a reason why they should never exhibit any, they may as well proclaim belief in any thing and every thing. The bottle conjurer may employ the same argument, and utter the same complaint—the directors of the aerial steam carriage company may consider themselves ill-used individuals—in short, every description of humbug must be welcomed with a bow, and no questions asked.

This is one of the fallacies with which enthusiasts deceive themselves. Because new truths have met with opposition on their first promulgation, therefore every thing new that is opposed is true, and all opposition to what is new is bigotry. The persecution of Galileo has been the ruin and the consolation of many a projector—the neglect of Milton has been the undoing of many a rhymester—and the discredit shewn to Harvey has comforted, if it has not generated a host of theorists. This is the way in which the followers of Johanna Southcote, of Hohenloë, and of Irving, steeled themselves against the derision of the world. The Saviour was mocked—so were they; their mission was, consequently, as divine as the Saviour's. The moralist may deem this a happy dispensation, the very hopelessness of the delusion constituting its alleviation; and perhaps, after all, a fool's paradise is not a bad one.

If the Zoist hugs himself with the fond idea, that his science and himself will be famous by-and-bye, well and good. It is a comfortable hallucination, and he does right to make the most of it. These pleasures of imagination are probably all he will ever get; and it were a pity to deprive him of them. But, like his "science," they *are* imagination, and one airy nothing will dissolve away with the other.

We laugh at the folly, we pity the madness of the Mesmerists. Many of them are neither better nor worse than arrant charlatans—more are weak dupes of knaves or of themselves—and a few are sincere, high-principled fanatics, led away by a love of the extravagant and marvellous, and trying very earnestly, but with small success, to hold the eel of philosophy by the tail.

The Editors of the Zoist promise that:—

"They will bestow great attention upon the practical department of the two sciences which they have undertaken to cultivate, but at the same time will not be regardless of their vivifying power. They will not forget that man, Eternal Man, is the theme; and, while they survey his past history and point out how his best interests have been neglected, they will indicate the manner in which these two sciences may be made subservient to the great end in view,—the progressive improvement and increasing happiness of the race."

We should be sorry to dispute the sincerity of these declarations, nay, we do believe that these gentlemen are actuated by the feelings they profess. But however estimable such sentiments may be, and however we may respect an earnest aspiration for the improvement of mankind, we must recollect that such sentiments and such desires, if not regulated by a sound and cautious judgment, run into enthusiasm, visionary at the best, and, perhaps, dangerous. In science, there can be no compromise with truth. Whatever leads to its establishment must be encouraged, whatever deviates from it, however amiable the feelings of the individual, must be sternly and resolutley reprobated. For our parts we view Mesmerism as a ludicrous, if not a mischievous, delusion; and, without regard to persons, it should be laughed at and put down.

---

### ON THE TREATMENT OF CYNANCHE TONSILLARIS. By DR. ROBERTSON.*

Surprised at the number of deaths which occurred in cases of croup under the usual treatment, Dr. Robertson determined to try a different plan of which the following is a sketch.

If the child is stout, an antimonial emetic is to be instantly administered; if young, or weak, or of a delicate constitution, a large proportion of ipecacuanha is given with the tartrate. Warm water ordered to be in readiness for a bath, if required. The moment the emetic has ceased acting, a stimulating liniment is applied to the neck and upper part of the chest, by moistening a piece of fine flannel, lint, or cotton wadding with the stimulant. These liniments vary according to the appearance of the skin, age of the patient, &c. A very good composition is about equal parts of oil of turpentine, compound camphor liniment, and olive oil: if the child is young, less turpentine. Occasionally iodine or ammoniacal preparations are employed; or tincture of cantharides: mustard sometimes, but its effects on the skin are often severe. If it does not redden

---

* Lancet, May 27.

the skin and cause acute pain in from two to five minutes, it must be strengthened. When once it acts freely, a very short time will answer. The inflammation should extend from the top of the thyroid cartilage to the top of the sternum, two or three inches broad above, and wider below. As soon as small vesicles occur, and a very red skin, with the papillæ much enlarged, the rubefacient should be removed, and replaced by a light soft poultice of bread and milk, or water, and some oil or lard, which must be changed or cleaned occasionally. The patient to be soothed and pleased, by trying to play with it, instead of crying or being dull; the less use they make of the nasal organs the better; a little liquorice to keep in the mouth, and if the cough is troublesome, pectoral mixture, with a very small quantity of opiate preparation, either compound tincture of camphor or one of the salts of morphia. Care must be taken in administering these, not to give a very large dose, or any if there is not much irritation; the stimulation of the liniment is often of service. Soon after the stomach has recovered from the effects of the emetic, calomel is given and is repeated every two, four, or six hours, and in doses proportionate to the age and strength of the child, narrowly watching the gums. When the least swelling or redness of the gums or throat is observed, stop for some hours and watch its constitutional effects. The bowels to be freely opened with castor oil, rhubarb, tartrate of potass, or some such medicine. If the skin is dry and hot, a warm bath is a good application; but if the patient's surface is moist, and itself weak or languid, we must be cautious in its application, taking care to cover the body the moment he is taken out, and instantly dry it with towels. If the medicines and applications do not produce the desired effect, they must be changed and others of somewhat a similar kind administered. Should any other disease, local or constitutional, be present, or come on during the attack, it must be properly attended to; proper temperature, &c. being adopted all the time.

During convalescence, the greatest care and attention must be paid to the patient; no exposure to cold or damp or any sudden transition of temperature: the neck, chest, and feet to be kept warm, and the diet simple: air and exercise in the arms, walking, or in a carriage, according to circumstances.

Under this treatment, Dr. Robertson states that very few patients have been lost.

---

### On Polypi of the Rectum in Young Children. By Dr. Gigon.*

After relating six cases of this affection, three of which were under his own care, and three under the charge of his colleague, M. Brun, Dr. Gigon makes the following remarks.

*Nature of the Tumors.*—These are stated to be fleshy, red, resembling a large cherry deprived of its epidermis, and with a bleeding surface, suspended by a narrow pedicle or stalk. Cut in pieces, it was found to be fleshy, of variable consistence, but usually having about the firmness of a portion of liver. To the naked eye there was no appearance of vessels; nothing like fibres, the mass was compact; in one case, however, by the aid of the microscope, some vascular traces were discovered, while, in another, in which the disease had lasted for a long time, a well-marked fibrous disposition was seen. In other respects, there was no difference of aspect in the six cases, there was always a globular tumor, of blood-red colour, and varying from the size of a cherry to that of a nut.

The pedicles were membranous, smooth, soft, of a greyish colour, and of the size of a small pea: they did not appear to be at all vascular, and their implant-

---

* L'Expérience, June 1st 1843.

ation took place at a height varying from some millimetres to six centimetres above the anus; they were insensible and endowed with but moderate powers of resistance; in one of these cases, the pedicle was not sufficiently firm to support the ligature, but broke, without giving rise to any consecutive hæmorrhage; this would, at first sight, appear to prove that the pedicle was not vascular, and that the discharge of blood which so often accompanies this disease comes from the rectum irritated by the presence of the polypus; this was, however, afterwards shewn not to be the case, because in one instance, after applying a ligature to the polypus, blood no longer exuded, notwithstanding the presence of the tumor in the intestine, and, in another, the excision of the pedicle was followed by severe hæmorrhage, proving that it is to the vessels which traverse the pedicle that the sanguineous exudations and hæmorrhages are due.

Two other species of polypous tumors of the rectum in infancy have already been noted, viz. mucous tumors, formed by the mucous membrane of the rectum, strangulated and distended, as observed by M. Stolz; vegetant tumors, as pointed out by M. Boyer, and, finally, fleshy pediculated tumors, as shewn in the present paper.

*Symptoms and Signs.*—The commencement of these tumors is attended with much obscurity; the rectal mucous membrane with too little sensibility to allow a body of such small size to occasion any sensation whatever. After some time, when the tumor has become well developed, the symptom most constantly observed is, an exudation, or even a flow of blood by the anus; the fæcal matters are then stained, and sometimes bathed in, and softened by pure blood, without any admixture of mucus. When this bloody discharge exists, and especially when it has continued for some time, without the presence of any general symptom, and without there being any reason for referring to some severe lesion of the intestine, the parents ought to be asked whether, when the patient goes to the water-closet, any reddish body appears at the orifice of the anus; if this has been observed, there is no longer any doubt, the nature of the disease is known; if not, the patient may be made to strain, the tumor will then usually project externally.

In some cases, in consequence of the shortness of the pedicle, or from its being attached very high up, the polypus cannot be brought into view, the finger may then be introduced without any difficulty, even in very young children, or the speculum ani may be employed.

The diseases with which this affection may possibly be confounded are, dysentery, hæmorrhoids, and prolapsus of the rectum.

From dysentery it may be distinguished by the absence of glairy matter, of abdominal pain, fever, heat, and, in fact, of every symptom with the exception of the presence of loose fæcal matter stained with blood.

From piles it may be distinguished by the colour of the tumor and its place of insertion; the age of the patient will also serve as a diagnostic mark.

With prolapsus of the rectum, this disease has been occasionally confounded; on examining the part, however, you find in prolapsus an opening in the centre of the tumor by which the fæcal matter escapes, whilst in polypus, the fæces escape by the side; in fact, a careful examination can leave no doubt upon the subject.

Supposing these tumors left to themselves, what would happen? Would they attain an indefinite size? M. Gigon is of opinion that they would not. The largest which he has seen was about the size of a nut, and in that case the discharge of blood had existed for upwards of a year. In some cases, these tumors may be detached spontaneously; the pedicle being ruptured during the passage of hardened fæces, or the polypus may be protruded, and finally separated by the action of the sphincter. However, the disease is one which ought not to be neglected, especially when the discharge of blood is abundant, as emaciation, hectic fever, marasmus may ensue.

*Treatment.*—The treatment to be employed in these cases, is at once simple and successful; the ligature must be had recourse to with or without section of the pedicle. Nothing is more simple than this little operation. The little patient is placed on his stomach, and made to strain till the tumor projects externally, it is then seized with a pair of forceps and drawn outwards; this may be easily done, for the mucous membrane is so lax at this age that very slight traction will enable you to get at the pedicle. When the polypus is thus drawn out it is somewhat difficult to distinguish where the pedicle ends, and where the mucous membrane begins, on account of the colour being the same; it is necessary, however, to pay some attention to this point in order that too much of the mucous membrane may not be included in the ligature, as this would be attended with inconvenience. A waxed thread is then passed round the pedicle, but must not be tied very tightly for fear of cutting through the pedicle. It is better then to return the whole into the rectum, without cutting it off below the ligature, as in one case in which excision was practised some hæmorrhage took place subsequently. The tumor left to itself in the rectum falls off on the second or third day, and is discharged at stool. In none of the cases observed by M. Gigon, was there any pain from the application of the ligature, or any accident after the operation. On the contrary, all the bad symptoms, such as the discharge of blood, loss of appetite, emaciation, &c. speedily disappeared.

---

### Climate of Van Dieman's Land.*

Van Dieman's Land, like most newly-colonised countries, has been represented as, in climate, an earthly paradise, and lauded as enjoying immunity from that great scourge of the human race—consumption. Mr. Power, Assistant-surgeon, 5th Dragoon Guards, has resided in the colony for three years, and visited every part of it where settlement has been made. He observes:—

"Van Dieman's Land is situated between the degrees, 41° and 44° south latitude, and 144° and 149° east longitude. This situation, though in fact ten degrees nearer the equator than Great Britain, affords a very similar climate in point of temperature,† to that which is experienced in the latter place; for, though more immediately under the influence of the sun's heat, the effect to be expected from such a locality is counter-balanced by the winds, which blowing uninterruptedly over the ocean from the perpetually frozen regions of the south, arrive there, without having their coldness modified, as occurs in the northern hemisphere, by a transit over large tracts of land. In this way, a medium temperature, a kind of balance as it were, is established, and extremes of heat or cold seldom continue for more than a very short period.

"This constant struggle between the cold winds of the south, and the heated air of the land, constitutes the premises from which has been inferred the great equability of temperature supposed by strangers to exist in that country; but, though the inference is just in general terms, experience proves that it is not so correct when examined more in detail. Thus it may be observed, that between the *different seasons*, the variation is comparatively trifling, the thermometer

---

* Dublin Journal, March, 1843.

† The thermometer, one season with another, ranges from about 42° to 74° in the shade; but when a hot wind blows, it sometimes rises suddenly as high as 108° or 110° in the shade.

frequently standing as high in winter as it does in summer, and *vice versa;* but at *all* seasons, the residents, particularly those living near the coast, are liable on *the same day* to experience the extreme heat of summer and the cold of winter; the change also frequently taking place, with a rapidity and suddenness which defies preparation, and is consequently extremely trying to invalids, or those who are of a naturally delicate constitution. This change is produced by the prevalence or otherwise of the sea-breeze, and its greater or less intensity; and the latter is again dependant on local causes, the consideration of which, it is deemed unnecessary to enter upon here."

Mornings and evenings are the coldest portions of the 24 hours. In summer about 10 or 11 a.m. the heat may be oppressive. In a single hour, there may be a cold sharp sea-breeze, searching the body, and setting up new, or reviving old rheumatisms. In an hour or two, the sea-breeze may again die away, and the close sultry weather return. Finally evening brings another change, unless, which is rare, there is a hot wind. These extremes are not felt so much in the interior of the country. The hot winds seem to be confined to the spring and commencement of the summer quarter, and are mostly from the north or north-west. They are usually mild, but occasionally one blights everything over which it passes. They seldom last more than a few hours, when a heavy fall of rain follows.

Snow never lies in the valleys—the slight frosts never survive the morning. The atmosphere during nearly the entire year is clear and the air rarefied. Animal spirits, are consequently exhilarated.

Pulmonary consumption, though not rife, *does* shew itself in Van Dieman's Land. It has committed havoc with the aborigines. Speaking of persons who had the seeds of the disease formed before leaving Britain, Mr. Power observes:

"It is worthy of remark, however, with respect to the latter cases that so far from being benefited by the change of climate, voyage, &c., to which they have been thus subjected, the course and development of the disease seems to be considerably accelerated, and a fatal termination to ensue at a much earlier period than would probably have occurred had the sufferer remained quietly at home. This point is mentioned chiefly because sea voyages have been recommended, on very high authority, in incipient or threatened cases of phthisis pulmonalis; no doubt this advice was intended to apply only to voyages made in the temperate zones, and the effects of a tedious voyage, no matter how perfect the accommodation on board, through the high latitudes south of the Cape of Good Hope, where during the greater part of the year the weather is cold, wet, and foggy, were not contemplated."

Hooping-cough, and the exanthemata present themselves, but mildly. The climate *may* assist female fecundity. Rheumatism is common and obstinate. The disease that has proved most fatal, of late years, is fever. From its peculiarities, it has received the name of "the Colonial Fever."

"Its commencement is very insidious, and all the symptoms, at that stage, extremely mild. If allowed to run its course without treatment, it not unfrequently happens that the fever may be ten days or more in existence, before the patient or his friends become aware of the serious nature of his indisposition; after that period, however, the accession of alarming symptoms is in general very rapid. As in typhus, the tongue becomes black, dry, and shrivelled; but the occurrence of petechiæ, or similar eruptions, is rather rare than otherwise. Head symptoms mostly set in very early in this stage, and are frequently complicated with such derangement of the bowels, as would lead to the supposition that extensive organic disease existed in the intestinal canal. In the majority of cases, however, where *post mortem* examinations have been made, the traces even of ordinary inflammation, in those situations, have been found to be so trifling, as to afford by no means a satisfactory explanation for the symptoms existing during life. But, the point to which perhaps the stranger's attention should be most impressively directed, is the frequent occurrence of relapses, and the extreme danger which attends them. Some time after the fever has apparently quite subsided, convalescence been well established, and the patient gradually gaining strength, it may be observed that the pulse still remains five or six beats in the minute, more frequent than natural. Under ordinary circumstances, this, viewed merely as a symptom of debility, might cause little uneasiness. In the colonial fever, however, it is necessary to watch it with the closest attention, for the patient can never be considered safe so long as such a state of pulse continues."

---

### Notes on Urinary Diseases. By J. Aldridge, M.D.*

Dr. Aldridge offers some hints which may be worth taking.

### 1. *Portal System of the Kidney.*

Dr. Aldridge alludes to the researches of Mr. Bowman, on the Structure of the Kidney—researches which, if established, would prove a strong analogy between that gland and the liver—viz. capillary tufts on the terminal branches of the arteries—efferent vessels from those tufts—and a second set of capillaries between those efferent vessels and the veins. However ingenious these views may be, we confess that they do not carry conviction to our minds, and whether we look at the observations, the analogies, or the hypotheses of Mr. Bowman, we are alike unsatisfied. But let that pass.

### 2. *Lithic Acid Deposits.*

The amorphous, or powdery, lithic acid deposit, can often be only distinguished from the crystalline by the microscope, yet the distinction is important. A copious deposition of lithates from the urine is a thing of minor consequence, such deposits in general corresponding to a very concentrated urine, in which there is not only an excess of urates, but likewise of urea and lactic acid; this excess depending, not upon an increase of solid excretion, but most usually on a deficiency of water.

"Super-lithate of ammonia (which, I believe, forms the bulk of these precipitates) is much more soluble in hot than in cold water; and thus, when a concentrated hot solution is permitted to cool, the excess becomes deposited. If transparent urine of a specific gravity 1.018 be evaporated gently to 1.028, and then permitted to cool, a cloud of lithates will deposit; and it is plain that the effect would be precisely the same, if, instead of removing some of the water, that portion had never been added. Urine evaporated in the manner described, would have not only the lithates, but the urea, lactic acid, and other constituents, also proportionately increased. Now this is precisely the character of the urine, from which a sediment of lithates ordinarily subsides."

Dr. Aldridge shews that the disorders in which this condition of urine obtains are often of an inflammatory character. "We perceive them accompanying chronic dyspepsia, and other cases where we have reason to think there is a subacute inflammation of some part of the intestinal canal; we see them in fevers both continued and intermitting; in rhematism and gout; after a debauch in wine, &c.; in all these cases theory would lead us to suppose the existence of an irritation in the kidneys. Again we find lithic acid deposits in dropsies depending on diseases of the heart and liver; in excessive secretions from any surface, whether profuse perspiration or diarrhœa; thus I have known persons whose urine threw down lithates as long as they were confined to bed, which

---

* Dublin Journal, March, 1843.

disappeared when they were able to get up and walk about; and many persons perceive a deposit in their water during Summer, but not in Winter: now in all those cases of flux or dropsy, there exists a derivation from the kidneys. May we not conclude, that lithates are deposited from the urine, when the quantity of water secreted by the kidneys becomes diminished, either by an irritation or by a counter-derivation, in obedience to well-ascertained physiological laws."

The opposite state of urine to that already dwelt on furnishes important diagnostic indications. Dr. Aldridge examined a case, in which from the general symptoms and stethoscopic signs, he pronounced on the existence of acute catarrh. But his attention being directed to the urine, he found this very pale and transparent. He re-examined the patient and found she had only hysteria.

*Crystallised Lithic Acid*, mixed frequently with blood globules, in an albuminous and very acid urine, Dr. A. believes, with M. Rayer, to be evidence of gouty nephritis.

### 3. *Alkalinity of the Urine.*

Dr. Aldridge's account of the chemical changes whereby urine becomes alkaline is so simple that we quote it:—

"Urea is a compound of the radical of carbonic acid (carbonic oxide) with the radical of ammonia (amidogene = $N. H_2$); when urea ferments, water becomes decomposed, and its elements uniting with the carbonic oxide and amidogene respectively, carbonate of ammonia is generated. Part of the ammonia formed neutralizes the lactic acid, to which urine usually owes its acidity; another portion unites with the biphosphate of magnesia, forming *ammoniacal magnesian phosphate*, and if the fermentation proceeds to any considerable extent, free carbonate of ammonia, producing effervescence upon the addition of acids, exists in the liquid."

The ferment that gives urea the disposition to decompose is usually the extractive matter of the urine; but pus and mucus are more powerful ferments still. In a given case of alkaline urine, the practitioner would first inquire, of course, if the patient has been taking alkalies, earths, their carbonates, the salts of these bases with the vegetable acids, or if he had been salivated by mercury. He would then ascertain if the urine was retained in the bladder. Dr. Aldridge does not believe that the urine ever becomes alkaline from putrefaction in the bladder, unless when mixed with pus or mucus, the globules of blood, if they act at all, operating very feebly as a ferment.

When the urine is *secreted* either fairly acid, or neutral, M. Rayer thinks there is inflammation, either acute or chronic, of the cortical and tubular substances of the kidneys. Every opportunity has been seized, at St. Vincent's Hospital, of testing the accuracy of this opinion.

"Among numerous instances, I may allude to the following: A case of essential hæmaturia, under the care of Mr. O'Ferrall, in the course of which repeated attacks of nephritis occurred; these were diagnosed from the supervention of shivering, pains, and deep-seated tenderness in the region of the kidneys, tendency to vomiting, &c.; in each attack the urine became neutral, and cupping the loins as regularly restored its acidity, at the same time that the other symptoms were removed. Cases of obstinate stricture, in which the same phenomena frequently occurred: A woman who laboured under spinal disease accompanied by catarrh of the bladder, and alcalinity of the urine, was treated by cupping, followed by setons over each kidney; the urine ceased to be alcaline long before the purulent discharge was diminished. In this case it deserves to be noted, that when the urine regained its acidity, she could retain it much longer than before. Another woman whose urine was very alcaline and deposited a mortar-like sediment, was examined after death; the bladder was the seat of numerous fungoid growths; but the kidneys were atrophied, firm, very adherent to their tunics, very pale in some parts, in others with greenish-brown depressions, at which situations the cortical substance was absorbed, the bases of the tubular fasciculi being in contact with the fibrous tunic: these are the appearances described by M. Rayer as occurring in subacute nephritis. In fine, the weight of evidence which has presented itself to us at St. Vincent's Hospital, has fully borne out the truth of the proposition advanced by the eminent pathologist I have mentioned."

Dr. Aldridge has found the fluid in the vesicles of herpes, and in the bullæ of pemphigus invariably alkaline.

### 4. *The Phosphatic Diathesis.*

Dr. Aldridge observes that this complaint has been admirably described and lamentably misunderstood. It has been considered as a result of cachexia; its essence to consist in a super-secretion of the urinary phosphates; and its treatment in the regulation of diet, the exhibition of opium, and the encouragement of *hope*, a hope too seldom realized. But Dr. Aldridge is prepared to show, that, in place of there being an increased secretion of the phosphates in this disease, the quantity of these salts in the urine is for the most part diminished. Dr. Aldridge presents us with a Table substantiating this position. We find, he says, by it that in place of being increased, the quantity of phosphates in the urine is actually, generally, diminished in the so-called phosphatic diathesis. The essential character of this kind of urine is not an excess of phosphates, but a neutral or feebly acid condition; and the cause of its tendency to alcalinity depends upon an existing subacute nephritis.

"A young man," he continues, "has stricture, or an old man has enlarged prostate or calculus, and constant retention, or friction, produces inflammation of the urinary passages; and this inflammation spreads along the ureters to the kidneys, to their cortical and tubular substances; and in consequence of this inflammation of the secreting substance, the urine becomes alcaline. Well, this spread of inflammation may be accompanied with fever, and frequent rigors, tendency to vomiting, &c.; and the case is called one of urinary fever; or, it may go on slowly, the fever of a hectic character, some deep-seated tenderness in one or both renal regions; weakness approaching to paralysis of the inferior extremities, much emaciation, and low spirits; and the patient is said to labour under the phosphatic diathesis. However, in both cases, the real disease is a *nephritis*—in the first, acute—in the second, subacute. In the former, the urine besides being neutral or alcaline, and depositing phosphates, is high-coloured, containing blood, and often very scanty. In the latter, this secretion is pale, and is frequently more abundant than natural. But in both, the alcalinity is the essential character of the urine, and not the circumstance of its containing phosphates in suspension.

"It is absurd, therefore, to talk of this as phosphatic urine, and of the disease which it signifies as a phosphatic diathesis.—The disease, so called, is none other than subacute nephritis, and the urine which accompanies it is characterized not by an increased secretion of phosphates, but by an essential alcalinity, or at least neutrality, of this liquid."

We have given Dr. Aldridge's sentiments on this matter at length, became they agree perfectly with our own. Without pretending to argue the chemistry of the matter, we have long found practically that persons labouring under alkaline urine and what is called the phosphatic diathesis were frequently not benefited by the tonics and mineral acids recommended for it, but not unfrequently made worse. In such cases we have seen *low* diet, abstinence from every thing stimulating, whether in medicine or in drink, and gentle laxatives produce marked benefit. We cannot say that, in any of these cases, we have tried depletion, or even counter-irritation, but we should have no hesitation whatever to resort to one or the other, when pain in the back, or other symptoms, seemed to offer a reason for it. We are glad, then, to meet with these observations of Dr. Aldridge's, because we believe that they are calculated to do away with injurious and routine practice.

But we must confess, that the alkaline diathesis, though often unimproved by tonics or by stimulants, will not bear with impunity *much* lowering. It is a nice point to steer between extremes, both bad, and the individual case must be watched, and the judgment of the practitioner exercised.

To return to Dr. Aldridge:—" I do not mean to deny, however, that there may be sometimes an increased quantity of the phosphates, occasionally present in disease: on the contrary, I have observed such to be the case, under certain circumstances. First, in old diseases of the bladder, such as ancient catarrh, fungoid growths, &c., there is frequently observed a deposit like mortar, consisting commonly of glairy mucus, and phosphate of lime, in enormous quantity. Now, when we recollect that phosphate of lime is contained in the urine, normally, in very small quantity, that it is notorious that calculi, composed of this substance, frequently form in the substance of the prostate, and that it is in the cloacum of birds (an organ analogous to the urinary bladder), that the egg acquires its shell, it is not, I conceive, too bold an hypothesis to imagine, that in these cases, the phosphate of lime is secreted by the lining membrane of the bladder.

" Secondly, I have seen some cases where crystals of the neutral ammoniacal magnesian phosphate were deposited in considerable abundance from an acid urine. The most remarkable example of this kind occurred in a boy, seven years of age, at St. Vincent's Hospital, who had rickets, and whose thigh was broken by a slight accident; the quantity of phosphoric acid contained in four ounces of his urine, was 8.76 grains: the fracture took long to reunite."

5. *Diagnostic Characters of Urine in Typhous Fever.*

There are generally met with three varieties of urine in typhous fever:—

1st. A pale transparent urine, of medium specific gravity, and possessing the ordinary properties of healthy urine.

2nd. A very deep-coloured transparent urine, of high density, passed in small quantity, and neutral in its re-action.

3rd. A deep-coloured scanty urine, transparent while warm, but depositing copious lithates according as it cools, and acid in its re-action.

" Of the foregoing, the first variety is rare, and is frequently considered, by practical physicians, a ground of unfavourable prognosis, inasmuch as it displays a want of balance in the febrile movement, for the same reason as a clean tongue in typhus is regarded by some as a bad sign.

" Most practical men consider the second variety as still more unfavourable, especially if it persist throughout the course of the fever. If, however, after a certain number of days, a cloud of lithates should present itself, or a lateritious sediment, as it is called, should fall down in the urine of a patient previously passing the second variety, this is considered a kind of fortunate crisis, and the patient's recovery is expected with greater or less confidence. In addition to this sign, some physicians regard the presence of albumen as an evidence of a favourable turn having taken place in the disease.

" Now let us contemplate the true nature of this high-coloured transparent urine. It is deeply coloured, because it is concentrated; being passed in small quantity, it contains comparatively little water, and therefore the colouring matter is not much diluted. It is transparent, because it is alcaline or neutral. The deposit, in the third variety I have described, consists of *super*-lithates. If aqua ammoniæ be added to this third variety, the sediment will re-dissolve, because the super-lithates, in that case, will be converted into neutral salts, which are soluble. Now the second variety being alcaline or neutral, the lithates are held in solution by the additional quantity of alcali present."

"This transparent kind of urine contains as much of the lithates as that which deposits a copious sediment. How futile, therefore, the hypothesis that an increased secretion of lithates is critical!

"This variety of urine is transparent, because it is neutral or alcaline. Now, an increased quantity of alcali may be present in the urine of typhus, from either of two causes; from the patient having taken some alcaline medicine, or from his labouring under a nephritic complication.

"Carbonates and citrates of the alcalies are amongst some of the most common palliatives employed in the treatment of fever. No data, for diagnosis or prognosis, can of course be drawn from urine, rendered transparent by the use of these medicines."

Dr. Aldridge proceeds to observe, that the urine may be neutral, or alcaline from nephritis, which may co-exist with typhus, independently of the symptoms usually said to indicate it. M. Louis gives one, and M. Rayer six cases of nephritis, complicating typhoid fever.

"In general, in these cases, there was the most marked prostration, dorsal decubitus, teeth and tongue covered with a sooty exudation, copious eruption; these patients had a constant tendency to stupor and muttering delirium, without much heat of head, contracted pupils, tendency to epistaxis; the urine was scanty, frequently suppressed, bloody, or albuminous, and alcaline; they all terminated fatally."

In cases of this sort the appearance of the lithates is a favourable symptom, not because a lateritious sediment is critical, but because it demonstrates the resolution of a dangerous complication.

It is the opinion of some that the presence of albumen is a favourable sign in typhus. Dr. Aldridge thinks that the phosphates have been taken for albumen. A *boiling* temperature throws down the former, but they are re-dissolved by nitric acid.

### 6. *Cerebral Symptoms produced by Renal Diseases.*

Every body knows, that interruption in the action of the kidneys leads to the accumulation of urea in the blood, and symptoms of cerebral oppression. But Dr. Aldridge makes a few remarks upon the subject.

The cases of disease, he says, in which the elements of the urine accumulate in the system, appear to depend either upon an obstruction in the kidney itself, or some of its ducts, or a derivation towards some other surface, from whence excessive secretion takes place. He is disposed to ascribe the retention of the elements of the urine in the blood in cases of calculus, clots of fibrine, or worms, impacted in some part of the passages, of mottled kidney, and of acute nephritis, to the first cause; and the suppression that occurs in cholera to the second.

But Dr. Aldridge does not clearly see why acute inflammation of the kidney should suspend secretion in it. This is contrary to the analogy of other organs, in which inflammation leads to an increase of secretion.

He alludes to a point connected with Bright's disease.

"In cases of granular degeneration, when the specific gravity of the urine is low, and this fluid is passed in a less quantity than natural, delirium alternating with stupor occurs, as a consequence of the accumulation of the essential elements of the urine in the blood. But it is not only in such cases that you have these symptoms presenting themselves in Bright's disease; occasionally, muttering delirium and drowsiness, are observed where the quantity of urine is so considerable as to prevent the supposition, that the accumulation can be dependant on a want of excretion. How are we to account for the cerebral symptoms under such circumstances?"

Dr. Aldridge alludes to the occasional supervention of head symptoms on the absorption of the effusion of dropsy from the cellular and serous cavities. Dr.

A. has never seen these symptoms shew themselves in dropsies from cardiac obstructions.

"Marchand and others have ascertained that urea exists in the dropsical effusions of Bright's disease. May not this fact afford us a clue to such cases as I have described? The dropsy becoming absorbed, the urea previously in the cavities is now thrown into the circulation and poisons the brain."

"Not only is accumulation of the elements of the urine in the blood produced by obstruction in the kidney or its ducts, but likewise by a derivation from the kidneys by excessive secretion elsewhere. A remarkable example of the operation of the latter cause occurs in spasmodic cholera. The copious rice-water discharges in this disease check most of the other secretions, and amongst the rest that of urine; urea consequently collects in the blood, and cerebral symptoms are produced."

Dr. A. suspects, as do we, that frequently urinary delirium and coma are mistaken for cerebral disease, a careless *post-mortem* examination soon strengthening the opinion.

### 7. *Air from the Urinary Passages.*

Dr. Aldridge relates a case of this description, communicated to him by Mr. M'Dermot.

The patient was attacked, in the Autumn of 1841, with excessive pain in the bowels, and was treated for inflammation; bled freely, and otherwise actively treated. He recovered with difficulty, and, on resuming his business (which required much and continued exertion), he became subject to the urinary disease. He stated, that it made its attacks after taking exercise, these being preceded by chill, rigor, and febrile disturbance. During the attacks he suffered from excessive pain in the region of the bladder that increased to agony when he attempted to make water; the urine was loaded, thick, and ropy, and threw down a deposit, which gave the idea of being feculent, and when he made water, there were, generally, one or two puffs of air from the orifice of the urethra, at the termination of the flow of urine. The urine had at times a very fætid odour. The man thought the air got into the bladder from the bowels, as, whenever he passed wind, he felt pain in the region of the bladder. He was, and looked in indifferent health. There was much pain on passing the catheter through the prostatic part of the urethra, but no distinct enlargement of the gland.

After taking some oxyde of silver pills, which disagreed very much, the complaint left the patient.

It is not very clear, whether there was a fistulous communication between the bladder and the rectum, or whether the air was not secreted by the inflamed mucous membrane of the bladder. The urine was that of chronic cystitis.

---

### ON THE INHALATION OF AMMONIA GAS AS A REMEDIAL AGENT. By ALFRED SMEE, Esq.*

If this gas is allowed to come in contact with the conjunctiva, it stimulates it, and causes much fluid to be poured from its secreting surface; when absorbed by the mouth in large quantities, it appears to cause in a similar manner an increase of the watery part of the secretion from all the parts which it reaches. The glottis offers no resistance to the passage of this gas when in a diluted state, but allows it to pass along the various ramifications of the bronchi, causing sensations which are extremely grateful and agreeable.

* Medical Gazette, April 7th.

"The immediate effect of the inhalation of this gas is to cause the fauces and phalanx, before dry, and perhaps covered with inspissated adherent mucus, to force out a watery fluid to lubricate and relieve the membrane; the phlegm will then separate and come away, and a more or less instantaneous relief is frequently felt."

The most convenient mode of administering it is, to use the vapour that spontaneously exhales from solutions of ammonia. For general purposes, the liquor ammoniæ of the shops, diluted with ten times its bulk of water, may be employed; this solution may then be placed in a common phial, and as much inserted as to occupy about the two lower inches of the bottle. The patient has then only to apply the mouth of the bottle to his lips, and draw in his breath, when he will inhale a certain quantity of the ammonia. The number of inspirations to be taken at one time may be regulated by the strength of the water and the effect of the remedy. Two, three, or four inspirations will, in general, be sufficient at one time, but this must be repeated three or four times during the day.

The vapour inhaled from the liquor ammoniæ does not seem to pass away immediately, but may be tasted for some minutes afterwards, even subsequently to the commencement of its beneficial action. The remedy is most applicable to cases of what is called dryness of the throat, which appears to arise from a deficiency of the proper lubricating fluid; the mucus becomes dry, and causes much uneasiness to the individual.

Ammonia gas is also beneficial in chronic hoarseness, especially in that which is often left as a sequela of influenza. It affords great relief to the relaxed swollen state of the mucous membrane, produced by remaining in crowded, overheated, and ill-ventilated rooms. In cases of incipient cynanche tonsillaris it appears to be of much value if used at the very commencement of the attack, the slight impediment to deglutition, which is generally the first premonitory sign, is sometimes removed by one or two inhalations.

"In old standing cases of asthma, especially in those in which the medical man considers that the internal use of the sesquicarbonate of ammonia is indispensable, in which the extremities are cold, the pulse feeble, and the general vital powers depressed, the local application of ammonia is particularly grateful, the patients feeling, as they describe it, a glow after its exhibition, and the warmth first imparted to the lungs extending by degrees over their whole system.

"In cases where the patient feels a peculiar sense of contraction upon passing into cold atmospheres, as though the lungs resisted the intrusion of so unpleasant an agent, the inhalation of ammonia seems to quiet the spasmodic action, relieve the breathing, and give a comfort to the whole chest, which is delightful to the feelings of the sufferer."

This remedy should, of course, not be employed during the presence of an acute state of inflammation, but in chronic cases, or when the circulation is feeble, the inhalation of ammonia may be used with the greatest advantage and comfort to the patient.

Ammonia is also stated to be useful as an antidote to certain direct and powerful poisons. One of these poisons, the effects of which it thus neutralizes, is bromine; the hurtful action of which is instantly counteracted by the vapour of ammonia. It is also useful when hydrocyanic acid is floating in the atmosphere of a room, as in this case it not only neutralizes the acid, but its stimulating properties are directly opposite to the depressing action of the acid.

In acting upon the lungs, and increasing their aqueous secretion, ammonia frequently causes at the same time a similar action on the skin by the exhalation of moisture from its entire surface. Hence during the prevalence of easterly winds, the gas may often be beneficially employed in determining to the surface and relieving the painful and annoying sense of constriction then experienced.

